# The influence of corticospinal activity on TMS-evoked activity and connectivity in healthy subjects: A TMS-EEG study

**DOI:** 10.1371/journal.pone.0174879

**Published:** 2017-04-06

**Authors:** Sara Petrichella, Nessa Johnson, Bin He

**Affiliations:** 1 Department of Biomedical Engineering, University of Minnesota, Minneapolis, Minnesota, United States of America; 2 Department of Computer Science and Computer Engineering, University Campus Bio-Medico, Rome, Italy; 3 Institute for Engineering in Medicine, University of Minnesota, Minneapolis, Minnesota, United States of America; University of Bologna, ITALY

## Abstract

Combined transcranial magnetic stimulation (TMS) and electroencephalography (EEG) can be used to analyze cortical reactivity and connectivity. However, the effects of corticospinal and peripheral muscle activity on TMS-evoked potentials (TEPs) are not well understood. The aim of this paper is to evaluate the relationship between cortico-spinal activity, in the form of peripheral motor-evoked potentials (MEPs), and the TEPs from motor areas, along with the connectivity among activated brain areas. TMS was applied to left and right motor cortex (M1), separately, at motor threshold while multi-channel EEG responses were recorded in 17 healthy human subjects. Cortical excitability and source imaging analysis were performed for all trials at each stimulation location, as well as comparing trials resulting in MEPs to those without. Connectivity analysis was also performed comparing trials resulting in MEPs to those without. Cortical excitability results significantly differed between the *MEP* and *no-MEP* conditions for left M1 TMS at 60 ms (CP1, CP3, C1) and for right M1 TMS at 54 ms (CP6, C6). Connectivity analysis revealed higher outflow and inflow between M1 and somatosensory cortex bi-directionally for trials with *MEPs* than those without for both left M1 TMS (at 60, 100, 164 ms) and right M1 TMS (at 54, 100, and 164 ms). Both TEP amplitudes and connectivity measures related to motor and somatosensory areas ipsilateral to the stimulation were shown to correspond with peripheral MEP amplitudes. This suggests that cortico-spinal activation, along with the resulting somatosensory feedback, affects the cortical activity and dynamics within motor areas reflected in the TEPs. The findings suggest that TMS-EEG, along with adaptive connectivity estimators, can be used to evaluate the cortical dynamics associated with sensorimotor integration and proprioceptive manipulation along with the influence of peripheral muscle feedback.

## Introduction

The functional activity of multiple areas of the brain acting simultaneously in concert is the basis for the performance of any behavior. The motor system, in particular, includes a network of several areas responsible for various stages of movement planning and execution. Transcranial magnetic stimulation (TMS) is a non-invasive brain stimulation method that can be used both to delineate and modulate brain function [1–6]. Simultaneous TMS and scalp electroencephalography (EEG) can be used to assess cortical reactivity and connectivity by directly stimulating a cortical region and evaluating the spatial and temporal propagation of the evoked activity. In this way, previous TMS-EEG studies have highlighted the promise of this combined approach, demonstrating the spatiotemporal spread of the TMS-evoked activity to a network of related cortical areas over time [7–9]. Former TMS/EEG studies of the motor network, in particular, have revealed that TMS-evoked activity spreads from the stimulation site to contralateral motor, sensory, pre-motor, and frontal areas over time [8,10–13]. Studies have also demonstrated that with increased TMS intensity, the peaks observed in the TEPs generally increase as well, with TMS typically being applied at supra-threshold intensities. Additionally, the quantity and timing of the spread of TMS-evoked activity from the stimulated area has been shown to be related to the state of the brain, with impaired spread during conditions such as sleep [7,14]. While former studies have provided a basis for understanding the expected topographical responses to stimulation of various brain areas, several questions remain unanswered.

TMS of the motor cortex evokes a series of activity within the corticospinal tract, causing a discharge in the spinal motor neurons and directly inducing the elicitation of Motor-Evoked Potentials (MEPs). It is well known that MEPs induced by stimuli of identical intensity and location can vary significantly in amplitude from trial to trial when recorded from a completely relaxed muscle [15–19]. With respect to the motor threshold, most TMS-EEG studies have evaluated either sub-threshold [7,9,10,20] or supra-threshold [8,12–14,21,22] stimulation and averaged all trials together, without respect to the MEP fluctuations. Thus, in comparing TMS-evoked potentials (TEPs) between supra-threshold and sub-threshold stimulation, the effects of increased TMS intensity on the evoked response cannot be separated from the effects of MEPs, as the likelihood of MEPs increases along with the TMS intensity. Applying TMS at motor threshold therefore presents a unique opportunity to study the effects of MEPs on TEPs, as MEPs are elicited in approximately 50% of trials by definition. No studies until now have directly evaluated the influence of the presence or lack of evoked corticospinal tract activity, measured in the form of MEPs, on TEPs without varying TMS intensity by applying TMS at the motor threshold.

Additionally, aside from topographical analysis, the dynamics among brain areas in response to stimulation remain far from well understood. Connections can be modeled by following approaches that include *a priori* hypotheses on the activity of the considered cortical areas (e.g., using dynamic causal modeling, DCM) [23] or data driven approaches such as Directed Transfer Function [24–26] or graph theory [27]. Importantly, the combination of TMS and high density EEG offers additional information regarding causality, providing effective connectivity (causal interactions) in addition to functional connectivity (temporal correlations) [7,25,26,28]. Connectivity analysis can provide insightful information on the inter-regional dynamics amongst brain areas over time, particularly in conditions known to be originating from a particular source, such as an epileptic source [27]. In a similar way, connectivity analysis can supplement traditional TMS-EEG analysis by providing dynamic spatiotemporal information on the strength and directionality of connections amongst a network of ROIs. While Granger Causality, and specifically Directed Transfer Function analysis, has been applied previously to a variety of datasets, such connectivity analysis has not yet been applied to TMS-evoked activity. When integrated into the traditional TMS-EEG analysis pipeline, Granger Causality-based connectivity analysis can capture dynamic relations between the stimulation target and associated brain areas.

The aim of the present study is therefore not only to evaluate the effects of cortico-spinal activity, measured as peripheral MEPs, on the resultant TMS-evoked activity but also to integrate connectivity analysis to reveal dynamic TMS-evoked connections within the motor network.

## Materials and methods

### Subjects

Seventeen healthy subjects (age range 18–58, mean 25, 12 female, 5 male) participated in the present study, which was specifically approved by the Institutional Review Board of the University of Minnesota. All participants were recruited between May 2013 and March 2015 and provided written informed consent prior to participation. The subjects received single pulse TMS to the left and right primary motor cortex (M1) while EEG was recorded. All participants were screened for any contraindications to TMS [29]. Any related medical history was reviewed and approved by a physician prior to participation.

### TMS

TMS was carried out by a Magstim Rapid^2^ stimulator and a 70 mm figure-of-eight coil. The coil was placed tangentially on the scalp at a 45° angle from the midsagittal line, approximately perpendicular to the central sulcus [30,31]. A Brainsight neuronavigation system (Rogue Research, Montreal, Canada) was used to verify optimal coil orientation to individual MRI data, localize the stimulation targets on the individual MRI of the subject, and estimate MNI (Montreal Neurological Institute) coordinates of the stimulation targets for each subject. The coil location and intensity were varied to determine the resting motor threshold (rMT), as the lowest stimulus intensity which produced MEPs ≥ 50μV in at least five out of ten consecutive trials [32,33]. After finding rMT for both left M1 and right M1 separately, the coil was stabilized and immobilized by means of a mechanical support. Magnetic stimuli were delivered at 100% rMT for each stimulation target, and the same intensity of stimulation was maintained throughout. The electromyography (EMG) response was continuously monitored for all subjects bilaterally from the first dorsal interosseous muscle using disposable EMG electrodes positioned in a belly-tendon montage and the Brainsight MEP Pod and recorded for 11 subjects. Each subject underwent an experimental session consisting of 2 blocks of 100 trials of TMS delivered at a frequency of 0.2 Hz, 1 block on the left M1 hotspot and 1 block on the right M1 hotspot. The order of stimulation targets was balanced across subjects. TMS was applied while subjects were seated in a comfortable armchair with their hands pronated in a relaxed position and eyes open. Subjects wore earplugs (Howard Leight Max, 33dB NRR, Honeywell, Morris Plains, NJ) for the duration of the experiment to reduce auditory activation during stimulation.

### EEG

TMS-compatible 64 channel EEG caps (Fast N’ Easy TMS Cap, Brain Products GmbH, Munich, Germany) were used along with TMS-compatible EEG amplifiers (BrainAmp MR Plus, Brain Products GmbH, Munich, Germany) with a wide dynamic range allowing continuous data recording without saturation of the EEG signals. Additional electrodes were used as ground and reference. The ground electrode was positioned in AFz and the FCz electrode served as the reference for all electrodes. The signals were bandpass filtered online at 0.1–500 Hz and digitized at a sampling rate of 5 kHz. Skin/electrode impedance was maintained below 10 kOhms for all subjects. Electrode positions were digitized and co-registered to each subject’s MRI by means of the Brainsight Neuronavigation System (Rogue Research, Montreal, Canada).

### MRI

Anatomical MR images were obtained for each subject using a T1-weighted magnetization prepared rapid acquisition gradient echo (MP-RAGE) sequence on a Siemens Magnetom Trio 3T Scanner (Siemens, Munich, Germany). Anatomical images were imported into the Brainsight Neuronavigation system and used to generate skin and curvilinear brain surfaces.

### Data analysis

Data analysis was conducted using MATLAB R2012b (MathWorks, Natick, Massachusetts, USA) and the public license toolbox EEGLAB [34]. For each subject, EEG was visually inspected for each trial in each channel and trials contaminated by environmental artifacts, muscle activity, or eye movements were rejected. Following this procedure, EEG signals were divided in segments of 3 s including a pre-stimulus baseline, stimulus artifact, and post-stimulus period. The segments between -1 to 0 sec and 8ms to 2sec (i.e. not including the stimulus artifact) were then low pass filtered at 80 Hz and notch filtered at 60 Hz using zero-phase second order IIR filters. All signals were then down sampled from 5000 Hz to 500 Hz and baseline corrected (100 ms pre-stimulus). For each electrode, all trials with EEG contaminated by values exceeding ± 200 μV from 20ms to 1s post-stimulus or ± 120 μV from 100ms to 1s post-stimulus were removed [35]. Rejected trials for each electrode were visually inspected and confirmed. EEG data for the included trials for each electrode and stimulation condition were averaged for each subject, and then averaged across subjects. Measurements of the amplitude and latencies of each component of the TEPs (using Cz as a reference for the peak component latency) were performed to obtain the topographical maps in the population. EEGLAB was used to produce potential maps at each latency corresponding to a peak in Cz. Additionally, for a subset of 11 subjects with recorded EMG data, the trials were divided into two sub-groups of *MEP* and *no-MEP*, based on the presence of an MEP of at least 50 μV or lack thereof, respectively. Statistical analyses were performed using paired t-tests between the potential values (μV) in the electrodes that corresponded to the peak response (>80% of maximum response) for each latency in the averaged evoked response across all subjects. The significance threshold of the results was fixed at *p <* 0.05, and false discovery rate (FDR) based correction was performed to correct for type-I errors.

### Source localization

Source localization was conducted for the TEP components using the eConnectome MATLAB toolbox [36] after the average of all trials and after the average of trials within the *MEP* and *no-MEP* sub-groups. The current density distribution of the averaged evoked response for all subjects in each condition was then projected onto the template MNI brain. Noise estimation (automatically calculated) was subsequently used to determine the sensor weighting and the regularization parameter (λ) of the current density reconstruction. The head volume conduction model was implemented using the Boundary Element Method [37,38] of the head having 3 compartments of fixed conductivities (scalp: 0.33 S/m; skull: 0.0042 S/m; brain: 0.33 S/m). Cortical current density imaging [39] was performed using the Minimum Norm Algorithm as implemented within the eConnectome MATLAB toolbox [36].

### Connectivity analysis

Connectivity analysis was performed using the adaptive Directed Transfer Function (aDTF) [40] within the eConnectome MATLAB toolbox [36]. Connectivity was calculated amongst regions of interest (ROIs) with centers located at the maximum of the current density activation corresponding to each peak of the TEP for channel Cz with a radius equal to 10 mm, in the time interval between 16 and 300 ms. A Multivariate Adaptive Autoregressive (MVAAR) model was constructed and used to describe the dataset composed by the data vector, model coefficients, and white noise, as described previously [40]. A Kalman filter algorithm, which describes the behavior of the multivariate signals by the MVAAR model, was used to determine the matrices of model coefficients over time. The time-varying modeling enables instantaneous calculation of the model parameters [41]. In order to prevent time-locked coupling among the estimated cortical activity in the ROIs and provide a statistical evaluation for the connectivity results, phase shuffling was performed 1000 times, as described in prior studies [40,42].

## Results

For the population of 17 subjects, the average rMT for left M1 was 58±10% of the maximum stimulator output (MSO). The average rMT for right M1 in the population was 60±13% of the MSO. For the 11 subjects with recorded EMG activity, the peak-to-peak mean MEP value for left M1 TMS was 289 ± 152 μV, while the mean MEP value for right M1 TMS was 225 ± 143 μV. After rejection of contaminated channels and epochs, 89% or trials remained for left M1 TMS and 91% for right M1 TMS. After dividing the trials into the *MEP* and *no-MEP* conditions, the peak-to-peak mean MEP value for left M1 was 402±243 μV for *MEP* trials and 16±3 μV for *no-MEP* trials. For right M1 TMS, the peak-to-peak mean MEP value after division was 429±196 μV for *MEP* trials and 16±3 μV for *no-MEP* trials. After subdivision, the proportions of *MEP* and *no-MEP* trials for left M1 TMS were 49±19% (total of 542 trials) and 51%±17% (total of 558 trials), respectively. For right M1 TMS, the proportions of *MEP* and *no-MEP* trials were 56±25% (total of 619 trials) and 43%±25% (total of 481 trials), respectively. Overall, the mean latency of MEP responses in the EMG was 23.7±2.1 ms for left M1 TMS and 23.3±2.0 ms for right M1 TMS.

### Overall TEPs

**Fig 1:**
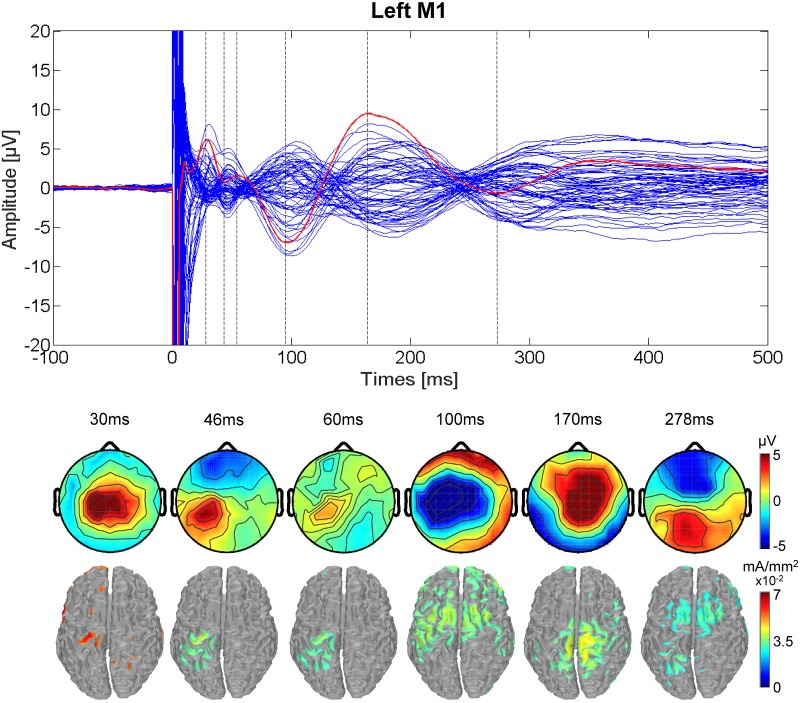
TEPs for left M1 stimulation. (Upper) Butterfly plot of the average TMS-evoked activity from all electrodes (average of all 17 participants). Red line indicates Cz electrode. Timing of the peaks is indicated by vertical dashed lines. (Lower) Voltage distributions (top) and Minimum Norm estimates (bottom) of the TMS-evoked activity for each peak in the Cz waveform. The cortical current density estimates have been thresholded to show the maximal activity at each peak.

Single pulse TMS of both left and right M1 evoked EEG activity lasting up to 300 ms [9,43–45] composed of a sequence of deflections of negative and positive polarity peaks, as reported previously in the literature. TEPs resulting from left M1 stimulation are shown in Fig 1. The stimulation resulted in positive peaks in channel Cz at 30, 60, and 170 ms post-TMS, and a series of negative peaks at 46, 100, and 278 ms post-TMS. The scalp topographies, along with the cortical current density estimates, shown in Fig 1, illustrate the spatio-temporal evolution of TMS-evoked activity. With respect to the cortical current density estimates for left M1 TMS, the maximum current density is in the left precentral gyrus corresponding to the hand area of M1 (Brodmann Area (BA) 4) at 30, 46, 60, and 170 ms after the stimulation, followed by the superior frontal gyrus corresponding to premotor cortex (BA 6) at 100 and 278 ms following the stimulation.

**Fig 2:**
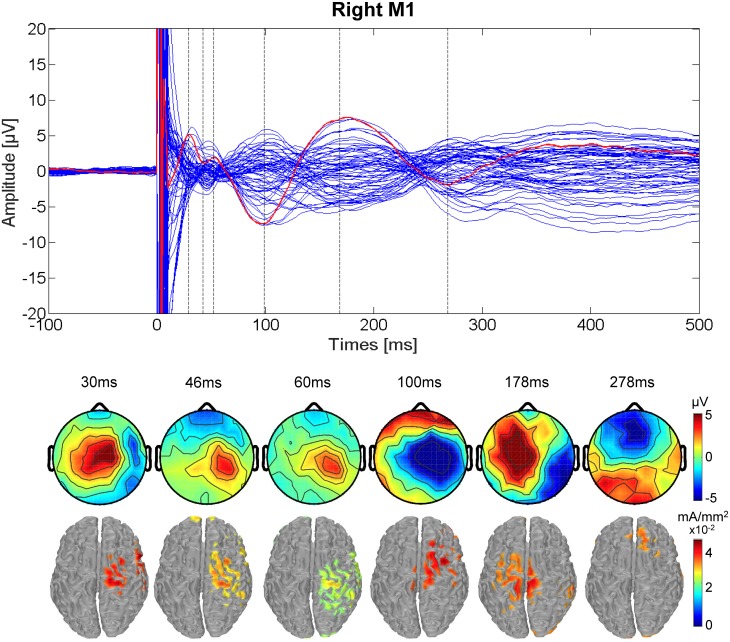
TEPs for right M1 stimulation. (Upper) Butterfly plot of the average TMS-evoked activity from all electrodes (average of all 17 participants). Red line indicates Cz electrode. Timing of the peaks is indicated by vertical dashed lines. (Lower) Voltage distributions (top) and Minimum Norm estimates (bottom) of the TMS-evoked activity for each peak in the Cz waveform. The cortical current density estimates have been thresholded to show the maximal activity at each peak.

The TMS-evoked activity resulting from right M1 stimulation is shown in Fig 2, including a consistent pattern of positive and negative peaks in the evoked response similar to that for left M1 stimulation. Cortical current density estimates indicate that the maximum current density progresses from the precentral gyrus hand area (BA 4) at 30, 46, and 178 ms, to the postcentral gyrus in somatosensory cortex (BA 3) at 60 ms, and the superior frontal gyrus in premotor cortex (BA 6) at 100 ms on the right side and at 278 ms on the left side.

### TEPs in MEP vs. no-MEP conditions

**Fig 3:**
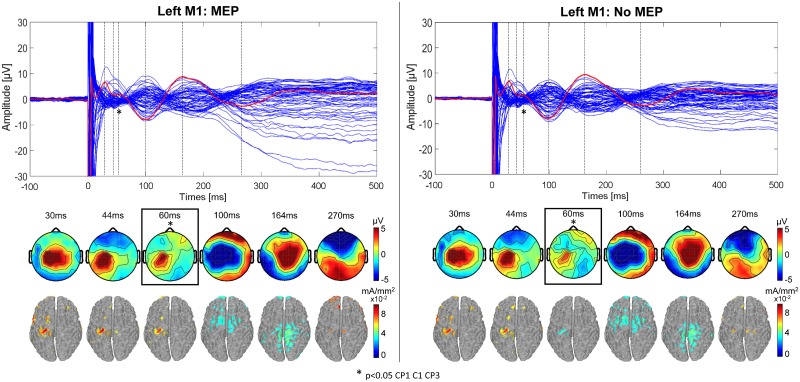
TEPs for left M1 stimulation for MEP and no-MEP trials. (Upper) Butterfly plot of the average TMS-evoked activity from all electrodes (average of 11 participants with simultaneous EMG recording) for trials with an MEP (left) and those without (right). Red line indicates Cz electrode. Timing of the peaks is indicated by vertical dashed lines. (Lower) Voltage distributions and cortical current density estimates of the TMS-evoked activity for each peak in the Cz waveform. The blue boxes and asterisks highlight the 60ms latency, for which the topography significantly differed between the *MEP* and *no-MEP* conditions for electrodes CP1, C1, and CP3 (*p<0*.*05*).

**Fig 4:**
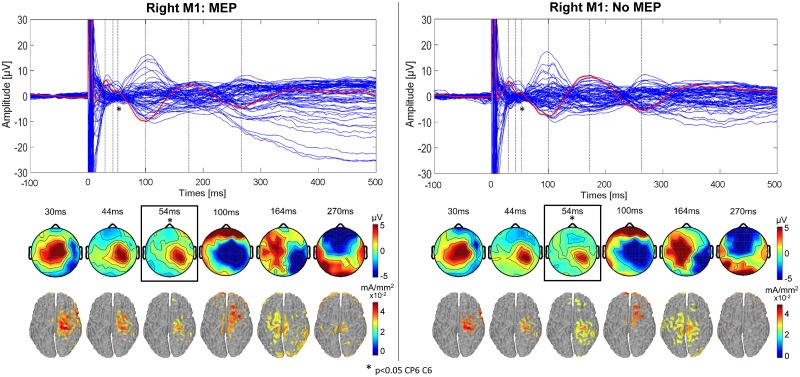
TEPs for right M1 stimulation for MEP and no-MEP trials. (Upper) Butterfly plot of the average TMS-evoked activity from all electrodes (average of 11 participants with simultaneous EMG recording) for trials with an MEP (left) and those without (right). Red line indicates Cz electrode. Timing of the peaks is indicated by vertical dashed lines. (Lower) Voltage distributions and cortical current density estimates of the TMS-evoked activity for each peak in the Cz waveform. The blue boxes and asterisks highlight the 54ms latency, for which the topography significantly differed between the *MEP* and *no-MEP* conditions for electrodes CP6 and C6 (*p<0*.*05*).

The excitability results for left M1 TMS are shown in Fig 3 for the *MEP* condition (left) and the *no-MEP* condition (right) at latencies of 30, 44 ms, 60 ms, 100 ms, 164 ms and 270 ms, respectively; similarly, the *MEP* and *no-MEP* condition results for right M1 TMS are shown in Fig 4 at latencies of 30, 44 ms, 54 ms, 100 ms, 164 ms and 270 ms, respectively. The topographical maps show a significant difference between the two conditions (*MEP* and *no-MEP*) at 60 ms for left M1 TMS in CP3 (p = 0.011, p_adj_ = 0.0195), CP1 (p = 0.035, p_adj_ = 0.035), and C1 (p = 0.013, p_adj_ = 0.0195) with p_crit_ = 0.035. Similarly, for right M1 TMS, a significant difference is shown at 54 ms in channels CP6 (p = 0.037, p_adj_ = 0.037) and C6 (p = 0.0026, p_adj_ = 0.0052) with p_crit_ = 0.037. For both stimulation conditions (left and right M1), the difference in amplitude (μV) is not only present in the motor hand area but also in the centro-posterior areas, with increased amplitudes in the *MEP* condition compared to the *no-MEP* condition. Source localization results indicate similar patterns of activation in the two conditions *MEP* and *no-MEP* for both left and right M1 TMS, with no significant differences between conditions.

### Dynamic connectivity results

**Fig 5:**
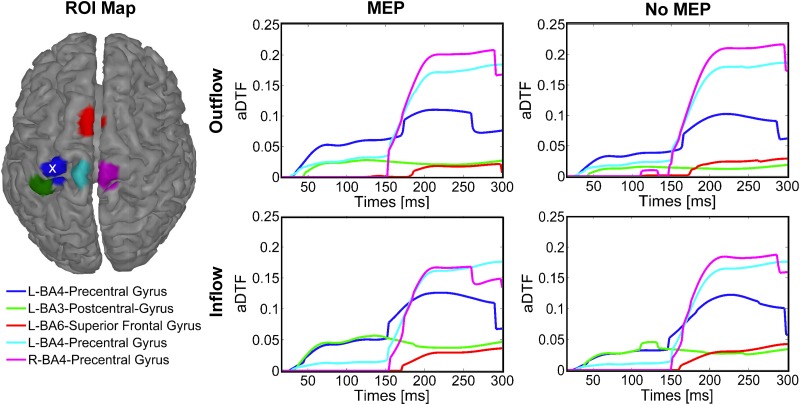
Inflow and outflow amongst ROIs for left M1 stimulation. Five ROIs were selected (left) using the cortical current density estimates for each latency (average of 11 participants with simultaneous EMG recording). The ROI corresponding to the stimulation target is indicated with a white x. Time-varying connectivity, as measured by the aDTF, was calculated for each of the five ROIs for trials containing MEPs (middle column) and trials without MEPs (right column), including both outflow (top row) and inflow (bottom row) patterns.

The connectivity patterns were calculated for each condition for the EEG data including all frequency content from 1–80 Hz. Connectivity analysis was also performed on individual frequency bands of the data for each condition (δ, θ, α, β, γ, not shown) and had similar connectivity patterns as those shown. The inflow and outflow connectivity patterns amongst the five ROIs are shown in Fig 5 for left M1 TMS. Fig 5 demonstrates that the ROIs corresponding to the stimulated area and BA3 have the highest inflow and outflow connectivity values in the time interval between 16 and 150 ms for the *MEP* condition. For the *no-MEP* condition, we observed a similar connectivity pattern with lower strength.

**Fig 6:**
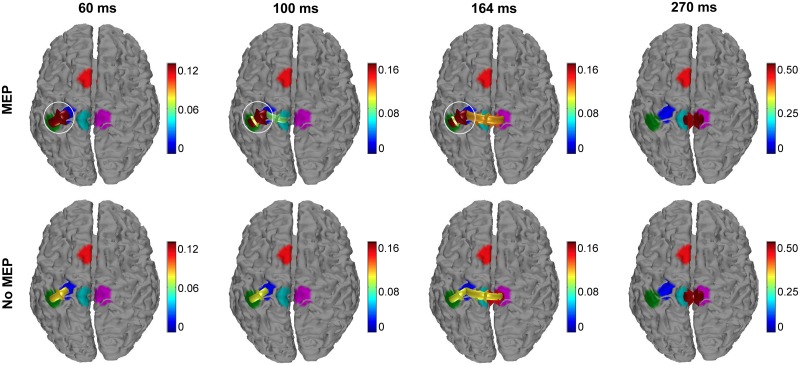
Connectivity patterns amongst ROIs for left M1 stimulation. Arrows indicate significant connectivity between ROIs at the 60ms (left), 100ms (middle left), 164 ms (middle right), and 270 ms (right) latencies (average of 11 participants with simultaneous EMG recording). The color of the arrow indicates the strength of the connection. White circles emphasize differences in connectivity patterns amongst trials with MEPs (top) and without MEPs (bottom).

The connectivity pattern obtained from the inflow/outflow time courses are shown in Fig 6 at latencies of 60, 100, 164, and 270 ms after left M1 TMS. At 60 ms, the connectivity directionality is from the left M1 toward the somatosensory cortex (S1) for both the *MEP* and *no-MEP* conditions, but with lower connectivity strength in the *no-MEP* condition. Importantly, a similar connectivity pattern is revealed respectively at 100 ms and at 164 ms between the *MEP* and *no-MEP* conditions, with a significant increase in the strength of the M1 to S1 connectivity in the *MEP* condition, along with the presence of connectivity from S1 towards M1 for the *MEP* condition only. At 270 ms, the connectivity pattern is the similar for the two conditions with connections reciprocally between the contralateral central BA 4 and the stimulated left central BA 4.

**Fig 7:**
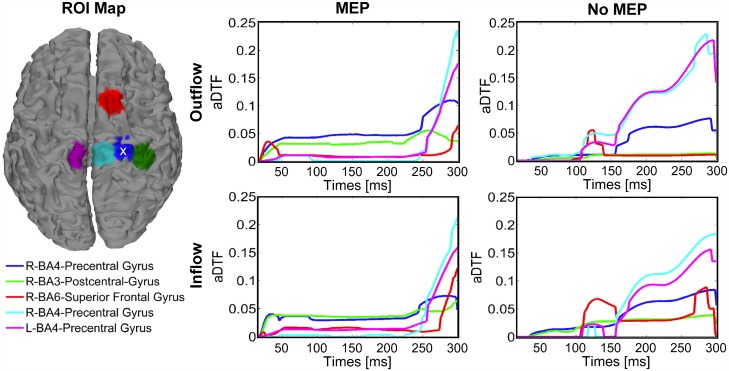
Inflow and outflow amongst ROIs for right M1 stimulation. Five ROIs were selected (left) using the cortical current density estimates for each latency (average of 11 participants with simultaneous EMG recording). The ROI corresponding to the stimulation target is indicated with a white x. Time-varying connectivity, as measured by aDTF, was calculated for each of the five ROIs for trials containing MEPs (middle column) and trials without MEPs (right column), including both outflow (top row) and inflow (bottom row) patterns.

For right M1 TMS (Fig 7), the outflow/inflow time courses showed similar patterns to those observed for left M1 TMS, with the highest outflow/inflow from the stimulated area and BA3 in the time interval between 16 and 100 ms and generally lower connectivity strengths for the *no-MEP* condition compared to the *MEP* condition.

**Fig 8:**
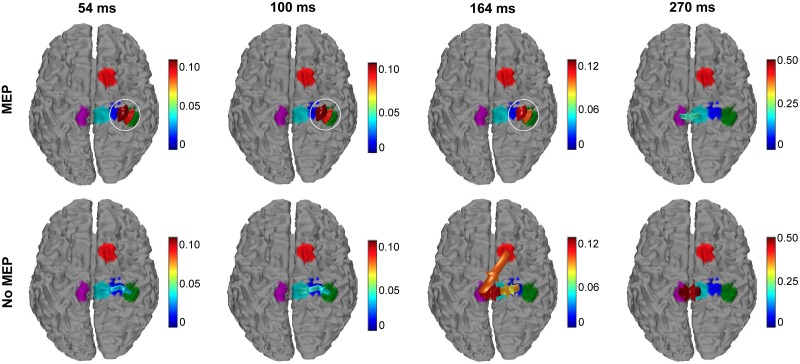
Connectivity amongst ROIs for right M1 stimulation. Arrows indicate significant connectivity between ROIs at the 54ms (left), 100ms (middle left), 164 ms (middle right), and 270 ms (right) latencies (average of 11 participants with simultaneous EMG recording). The color of the arrow indicates the strength of the connection. White circles emphasize differences in connectivity patterns amongst trials with MEPs (top) and without MEPs (bottom).

The connectivity patterns obtained from the inflow and outflow time courses are shown in Fig 8 at the latencies 54, 100, 164, and 270 ms after right M1 TMS. At 54 ms and 100 ms for the *MEP* condition, the connectivity pattern is mutual between right M1 and right S1. Importantly, for the *no-MEP* condition, the connectivity directionality is only from the right M1 to right S1, with significantly lower values in the strength of the connections. At 164 ms after the stimulation, the pattern for the *MEP* condition shows the same pattern observed at 60 and 100 ms, while in the *no-MEP* condition the connectivity directionality is mainly from the contralateral central M1 to the right supplementary motor area, and reciprocally between the right central M1 and both contralateral central M1 and the stimulated right M1. At 270 ms, the connectivity directionality pattern is the same for both the conditions between the contralateral central M1 and the right central M1 reciprocally, with higher connection strength in the *no-MEP* condition.

## Discussion

### Source localization: Origins of TMS-evoked activity

The present results demonstrate that the spread of the activations for both left and right M1 TMS is from the stimulated motor area to central motor and parietal areas, along with areas in the middle, superior and inferior frontal lobe. Overall, the timing and localization of evoked activity is generally consistent with that observed in previous studies including motor areas [46,21]. Regarding the origins of each latency, the activations shown within the stimulated area at 30ms and 44ms for left M1 stimulation are likely due to excitatory activity intrinsic to this area [46], though previous studies have suggested that the 30ms latency could also reflect the activation of subcortical pathways, specifically to the thalamus and basal ganglia, which also include reciprocal connections back to cortical areas [21]. Activation at a latency of 44ms could also be attributed to motor areas outside of M1, including pre-motor and supplementary motor cortex, as suggested in a previous study [21]. The 60ms latency likely reflects somatosensory integration, as the peak response in cortical current density is in BA 4, with an important spread towards BA 3, corresponding to the hand area of primary somatosensory cortex. This area has thalamo-cortical projections (to the ventral postero-lateral nucleus) and its function includes stimuli localization and stimuli intensity proprioception. Parietal areas, dedicated to the analysis of proprioceptive information for motor control [47], are also activated in later latencies suggesting further integration of somatosensory and motor information resulting from the TMS pulse in the TEP. The N100-P190 complex has been hypothesized, in previous studies, to be due in part to the sound emitted from the coil during stimulation [48,49]. However, in more recent studies, the cortical response in these latencies persisted as previously observed even when white noise was used to mask the sound of the coil click [8,46,50], suggesting that this late component can be caused at least partially by cortical activation of areas interconnected with the stimulated area [46,21]. Casula et al. [51] showed, as observed in this work, that the topographic distribution of the N100 latency was not in agreement with the distribution normally observed for a purely auditory component. Compatibly, we found that for left M1 TMS, the cortical current density maps at 100 ms and 278 ms reach the maximum in the left superior frontal gyrus (BA 6), corresponding to the premotor area, and in the precentral gyrus (central BA 4) at 170 ms. The left superior frontal gyrus, in particular, is known to be dedicated to high-order associative functions and to be part of the motor network. For right M1 TMS, the cortical current density distribution reveals a similar pattern compared to left M1 TMS, with presumptive common cortical sources for each latency. Despite the notion that the N100-P190 complex contains relevant cortical activation of the stimulated area, the possibility that these latencies are largely contaminated by auditory activation cannot be excluded. For example, a recent study [52] demonstrated that using ear protection alone during TMS reduces, but does not eliminate, the N100-P190 complex. However, as the primary purpose of this study was to evaluate the relationship between peripheral MEPs and TEPs, as a comparison between two conditions with equal experimental setup, the potential for auditory evoked potentials within TEPs was deemed acceptable.

Two studies have previously reported a defined pattern of activation following TMS to left M1 [8,10], in which TMS produced large deflections in scalp voltage primarily near the site of stimulation but also on the contralateral side. Huber et al. [10] reported activation for left M1 stimulation in one subject at 30, 80, and 120 ms following TMS in the same region of the stimulation along with a contralateral activation at 40 ms after the stimulation. Paus et al. [8] showed that, in a group of seven subjects, the mean evoked activity consisted in current density results for the left M1 TMS at 44 ms after the stimulation localized in the site of stimulation, as we reported. However, neither of these studies included right M1 TMS in addition, and both included fewer subjects and latencies than presently reported. Several other previous studies have included TMS-EEG for premotor cortex stimulation (REF), resulting in a similar series of deflections as reported in the left M1 studies and in the present study. In summary, we found that when averaging all trials together regardless of EMG response for each stimulation condition (left and right M1), the source localization from the site of stimulation (early latencies) spreads toward the somatosensory, premotor, and central motor areas in the later latencies in agreement with previously published studies.

### TEPs and effective connectivity in MEP and no-MEP conditions

A unique conclusion of the present study was that for both left and right M1, a significant difference was observed in the amplitude of the response in the somatosensory area near 60 ms between the *MEP* and *no-MEP* conditions in a subgroup of 11 participants. The notion that a difference was only observed at an individual latency, 60ms, is interesting given the dynamics of the evoked activity within a variety of areas. The slight, yet significant, difference in evoked potential strength at 60ms despite unchanging topographical patterns across conditions suggests that such a difference represents a smaller signal superimposed on the larger dominating evoked response. TMS-evoked responses are generated from the summation of both excitatory and inhibitory post-synaptic potentials, while MEPs are produced from brief descending action potentials originated by such post-synaptic potentials [32]. Therefore, it is possible that alterations in overall excitability pre-stimulus could result in trials with increased likelihood of MEPs and, subsequently, greater evoked response peaks. Previous studies have investigated the relationship between pre-stimulus activity and MEP size, demonstrating that coupling between activity in specific frequency bands is related to the likelihood of MEP elicitation [53]. Indeed, the generation of MEPs depends not only on the integrity of the cortico-spinal tract, but also on the intensity and timing of the stimulation along with the direction of the induced current [54]. In this study, we control for all of those potential influences with the exception of stimulus timing relative to ongoing cortical dynamics. However, if overall cortical activity level at the time of stimulation was the origin of the difference observed at 60ms, it remains unclear why only the 60ms latency was affected.

Alternatively, the difference observed at 60ms could potentially reflect afferent feedback from other areas. Specifically, this difference could be due to somatosensory feedback resulting from peripheral motor activation caused by TMS as part of a complex cascade of activity representing corticospinal tract activation [11]. In addition, it has been noted previously that MEPs can vary significantly in amplitude despite unchanging stimulation parameters, suggesting fluctuations in the functional state of M1 and the excitability of spinal motor neurons recruited through the corticospinal tract [17,21,55]. In the present experiment, it has been observed that the conduction time between the brain and small hand muscles is approximately 20–25 ms in case of M1 hand area stimulation. Somatosensory feedback resulting from the stimulation of the target muscle can therefore affect cortical responses measured 50–60 ms after stimulation [21,56]. Therefore it is possible that the presence or lack thereof afferent somatosensory information resulting from the peripheral MEP could explain, at least in part, the difference observed at 60ms following the pulse. This is supported by the connectivity results highlighting the significant difference in connection strength between M1 and S1 for the MEP and no-MEP conditions, highlighting the relevance of S1 in the MEP condition.

Previous TMS-EEG studies have demonstrated that with increased stimulation intensity, the overall amplitude of TEP components tends to increase, while maintaining the original pattern of cortical origins and polarity of peaks. However, as stimulation intensity increases, the likelihood of generating peripheral MEPs from TMS also increases, with potential implications to the TEPs. One other recent study has also begun to investigate the relation between peripheral muscle activation and cortical responses after TMS [12]. Giambattistelli et al. [12] investigated the relationship between TEPs and MEP amplitudes resulting from supra-threshold TMS, specifically considering regions of interest corresponding to activated cortical areas. In that study, they claimed that cortical activity ipsilateral and contralateral to the site of stimulation is correlated with the amplitude of MEPs in the target muscle. However, this study applied supra-threshold stimulation, resulting in MEPs in most trials, and separated low and high MEP conditions based solely on the distributions of the MEP amplitudes. Applying TMS at motor threshold presents a unique opportunity to study the differences between TEPs resulting from trials with MEPs and those without MEPs, without changing the stimulation intensity. In the present study, stimulation was applied at threshold, avoiding a statistical imbalance in the number of trials in each category, and our MEP and No-MEP conditions were determined by the standard definition of an MEP (≥50μV), not on the distribution of responses. Additionally, prior studies were conducted with suprathreshold TMS, in which high and low MEP values do not correspond to any relevant division per se (i.e. all of the MEPs are likely much more than 50 μV, even in the low category). The threshold of 50 μV is based on the standard and accepted definition of rMT in the TMS field, and was likely originally set as a reflection of measurable motor output significantly above the level or noise that could be attributed to TMS.

With respect to the connectivity analysis, this study was the first to integrate aDTF with TMS-evoked responses for the purpose of evaluating dynamic connections amongst ROIs activated via TMS. While Granger Causality approaches can be of great value for a variety of applications, the utility of such methods in perturbation-based imaging is particularly of great interest. The results of the present study demonstrated that with respect to the connectivity pattern, for both the left and right M1 TMS, the maximum outflow and inflow is in the stimulated area in the early latencies, and in contralateral and central motor areas in later latencies. Interestingly, while the pattern of connectivity was relatively stable between the *MEP* and *no-MEP* conditions, significant differences were observed in connection strength between the conditions, particularly between M1 and S1 on the stimulated side. The connectivity results for the *no*-*MEP* condition display overall lower values of outflow and inflow connectivity throughout the studied time interval, suggesting that the strength of connections between areas is correlated with MEP values. While differences between conditions are described qualitatively, it is important to note that only significant connections were included in the results presented. Based on the methods presented in [40], a random and independent shuffling of Fourier coefficient phases can be used to perform a nonparametric statistical test of aDTF values. The shuffling procedure was repeated 1000 times to create a null distribution of aDTF values, and the unshuffled aDTF values were statistically compared to the null distribution. In this way, the dynamic connectivity results were statistically evaluated for each condition (*MEP* and *no-MEP*) and comparing the significant connections across conditions qualitatively remains valid. The results of the present study highlight the utility of adaptive connectivity estimators for evaluating network-level interactions in response to stimulation.

While other studies have investigated the relationship between pre-stimulus activity and MEP amplitudes [13,57], no other studies, to our knowledge, have evaluated the influence of the presence—or lack thereof—of corticospinal tract activity (measured through MEPs) on the amplitude and distribution of TEPs or on the effective connectivity patterns of the correspondent current density maps. The present study is the first to include excitability, current density source imaging, and effective connectivity analysis in evaluating the influence of MEPs on TEPs. Additionally, our connectivity results showed a modulation between M1 and S1 connectivity ipsilateral to the stimulation between *MEP* and *no-MEP* conditions, in the latencies that follow the elicitation of MEPs, which may reflect somatosensory feedback, sensorimotor integration, and proprioceptive manipulation. The present results demonstrate that higher levels of corticospinal tract activity, measured through MEPs, correspond to higher levels of effective connectivity between M1 and S1 in a mutual way. Given the relationship between MEPs and the strength of the evoked response, in terms of topography and connectivity, these results suggest that future TMS-EEG studies should record and consider the influence of EMG activity on resultant EEG responses rather than averaging all trials together with varying EMG signals. Additionally, the present study highlights the utility of including connectivity analysis, in the form of aDTF in this study, for further characterization of the dynamics of evoked potentials following TMS.

Despite promising results, we acknowledge several limitations of the present study. The limited number of subjects, particularly those with recorded EMG used for the delineation of *MEP* and *no-MEP* conditions, may affect the reliability of the results. Future work incorporating additional subjects should be pursued to evaluate the stability of the observed differences in a wider population of subjects. Additionally, the data analysis procedure may have influenced the resultant evoked-activity without intent. The artifact rejection procedure, in particular, was performed for each channel and epoch independently prior to averaging. Thus, it is possible that the average evoked-responses for each channel resulted from a different number of epochs. Although this did not appear to influence the overall time course and topographical distribution of the evoked response in the present study, the artifact rejection could indeed have impacted the results. Similarly, we did not use independent component analysis or other artifact rejection means to remove other types of artifacts that can result from application of TMS, such as muscle artifacts. Given that such techniques can easily remove relevant portions of the signal of interest along with artifactual data, and that muscle artifacts generally occur before the time period of interest for the present study (30ms post stimulus and after), such muscle artifact removal was not pursued. However, it remains possible that muscle or auditory artifacts remain present in the data presented. Future work could incorporate additional artifact removal techniques to further validate the differences resulting from the elicitation of MEPs in response to TMS. Future work could also analyze components and latencies for each electrode independently, with the potential to further characterize the difference between *MEP* and *no-MEP* conditions. In a similar fashion, future work could incorporate statistical analysis on connectivity differences between conditions, with phase shuffling performed on differences over time between conditions. However, despite these challenges, this study establishes the feasibility and potential for integrating connectivity analysis into TMS-evoked EEG analysis and highlights the relationship between MEP elicitation and the TMS-evoked EEG response at 60ms.
